# Acute kidney injury as a risk factor for diagnostic discrepancy among geriatric patients: a pilot study

**DOI:** 10.1038/srep38549

**Published:** 2016-12-16

**Authors:** Chia-Ter Chao, Hung-Bin Tsai, Chih-Kang Chiang, Jenq-Wen Huang, Kuan-Yu Hung, COGENT study group, Chih-Yuan Shih, Su-Hsuan Hsu, Yu-Chien Hung, Chun-Fu Lai, Derrick Ding-Cheng Chan, Chung-Jen Yen, Tzong-Shinn Chu

**Affiliations:** 1 Department of Medicine, National Taiwan University Hospital Jinshan branch, New Taipei City, Taiwan; 2 Graduate Institute of Toxicology, National Taiwan University College of Medicine, Taipei, Taiwan; 3 Division of Nephrology, Department of Internal Medicine, National Taiwan University Hospital, Taipei, Taiwan; 4 Department of Traumatology, National Taiwan University Hospital, Taipei, Taiwan; 5 Department of Integrative Diagnostics and Therapeutics, National Taiwan University Hospital, Taipei, Taiwan; 6 Department of Internal Medicine, National Taiwan University Hospital Hsin-Chu branch, Hsin-Chu County, Taiwan; 7 Department of Gerontology and Geriatrics, National Taiwan University Hospital, Taipei, Taiwan

## Abstract

Diagnostic discrepancy, defined as different admission and discharge diagnoses, could be a potential source of diagnostic error. We evaluated whether acute kidney injury (AKI) in the elderly affected their risk for diagnostic discrepancy. Patients aged ≥60 years from the general medical wards were prospectively enrolled and divided according to AKI status upon admission, using the Kidney Disease Improving Global Outcomes (KDIGO) criteria. We compared their discharge and admission diagnoses and identified patients with a diagnostic discrepancy, using multiple logistic regression analysis to evaluate the relationship between initial AKI and the presence of a diagnostic discrepancy. A total of 188 participants (mean age, 77.9 years) were recruited. Regression analysis showed that initial AKI on admission was associated with a higher risk of diagnostic discrepancy upon discharge (odds ratio [OR] 3.3; *p* < 0.01). In contrast, higher AKI severity was also associated with an increased risk of diagnostic discrepancy (for KDIGO grade 1, 2, and 3; OR 2.92, 3.91, and 4.32; p = 0.04, 0.03, and 0.02, respectively), suggesting that initial AKI upon admission could be an important risk factor for diagnostic discrepancy. Consequently, reducing geriatric AKI might have the potential to reduce diagnostic discrepancy among these patients.

The number of elderly patients is increasing worldwide. Healthcare spending in this population is increasing, owing to multimorbidity, functional decline, a higher prevalence of frailty, and increased vulnerability to adverse environment^1^,^2^,^3^. Elderly patients who sustain acute illness, especially infection-related episodes, are more likely to be hospitalised than their younger counterparts; studies have identified a progressive increase in the incidence of hospitalization with older age, irrespective of pathogens^4^. Elderly patients undergo medical investigation and intervention frequently during their visit to the emergency department and during hospitalizations, leading to the increased utilization of healthcare resources^5^.

A prompt and accurate diagnosis upon admission is important for all patients, since a correct initial diagnosis facilitates the selection of the appropriate management strategy and thus influences subsequent treatment planning. A mismatch of diagnoses upon admission and discharge can have major clinical implications, as a diagnostic discrepancy might herald a higher incidence of diagnostic error and potentially increase the possibility of implementing unnecessary examinations or interventions^6^. Large practice-based registries have shown that diagnostic errors are responsible for 7 to 17% of in-hospital adverse events, contributing to increased hospital mortality^7^,^8^. Consequently, the identification of diagnostic discrepancy among hospitalised patients can pave the way toward uncovering diagnostic errors and improving the quality of inpatient care.

Currently, there are very few studies addressing factors influencing diagnostic discrepancy among the hospitalised elderly, despite available reports suggesting that increased age might be associated with a higher incidence^9^,^10^. We previously found that the elderly diagnosed with acute kidney injury (AKI) upon admission had a significantly higher risk of developing in-hospital complications and increased mortality^11^,^12^. As patients with renal disorders reportedly have a higher risk of a diagnostic discrepancy^9^, we hypothesised that the presence of an initial diagnosis of AKI upon admission in the elderly might also affect diagnostic discrepancy upon their discharge. We prospectively investigated the relationship between the presence of AKI and diagnostic discrepancy by utilizing a consecutively enrolled cohort of geriatric patients.

## Methods

### Recruitment of participants and study design

Patients aged 60 years and older who were hospitalised in the general medical wards of National Taiwan University Hospital (NTUH) between December 2013 and November 2014 were prospectively enrolled. NTUH, an affiliate of National Taiwan University, is a 2000-bed tertiary medical center located in the capital of Taiwan serving patients with acute and chronic care needs around the country^13^. There are six general medical wards in NTUH admitting patients with general medical diseases including urinary tract infection, pneumonia, ileus, and others, but are not limited to specific types of disease. Those with surgical diseases or those requiring surgical interventions are ineligible for admission to the general medical wards. The mean age of admitted patients is 66.4 years, with a mean Charlson comorbidity index of 3, and 53.4% are men^13^. Pneumonia is the most common admission diagnosis, followed by urinary tract infection; nearly 95% patients were admitted *via* the emergency department.

Upon admission of the patient, we recorded medical history, age, sex, a comprehensive list of comorbidities, and their vital signs (blood pressure [BP] and heart rate [HR])^11^,^14^. Comorbidities were ascertained based upon corresponding laboratory data, imaging findings, pathologic evidence, or by certified specialists as appropriate. Laboratory data including haemogram and serum biochemistry test results were also collected. Diagnoses at admission were independently made by the attending staff, and were categorised into cardiopulmonary disorders, nephrourological disorders, gastrointestino-hepatic disorders, haemato-oncologic disorders, fever of unknown origin (FUO), and acute cerebrovascular events^15^. Variables pertaining to treatment courses, including intensive care unit transfer and hospital mortality, were also recorded.

Presence of initial AKI was also ascertained upon admission, according to the serum creatinine (Scr) criteria of the KDIGO classification^16^. In brief, AKI was diagnosed if, upon admission, the participants had an Scr level increase ≥0.3 mg/dL within 48 hours or a 50% increase from their baseline Scr level within the 7 days before the current admission. In addition, grade 1, 2, and 3 AKI were diagnosed if the Scr level increased 50%, 100%, or 200% from the baseline level, respectively. We retrieved the baseline Scr level of all participants from three months before and up to the time of their admission. Chronic kidney disease (CKD) was defined as a baseline estimated glomerular filtration rate (eGFR) of less than 60 ml/min/1.73 m^2^ according to the Chronic Kidney Disease Epidemiology Collaboration (CKD-EPI) formula.

This study was approved by the ethical committee of NTUH (No. 201306089RINA) and all participants provided informed consent. This study adhered to the tenets of the Declaration of Helsinki.

### Outcomes of interest

All elderly patients were prospectively followed up until discharge from the hospital or death. The same staff provided the admission and discharge diagnoses. After patient discharge, we documented discharge diagnoses using a similar classification method, and compared the discharge and admission diagnoses. A diagnostic discrepancy was noted if the main discharge and admission diagnoses differed.

### Statistical analysis

SPSS 18.0 software (SPSS Inc., Chicago, IL, USA) was used in this study for statistical analysis. We described continuous variables as the mean ± standard deviation. Comparisons between each group were performed using an independent *t*-test or a Mann-Whitney *U* test, as appropriate. We described categorical variables as event numbers and percentages, and between-group comparisons were performed using a chi-square test.

Initially, we examined the overall clinical features including demographic profiles, comorbidities, admission diagnoses and vital parameters, as well as laboratory results. After these patients were discharged, we collected their discharge diagnoses to ascertain whether there was a diagnostic discrepancy. Those with diagnostic discrepancy after hospitalization are described in detail. We then compared clinical data between the participants with and without AKI to establish any significant differences. On univariate anlaysis, we made comparisons between those with and without diagnostic discrepancy. Those with significant differences were then selected and subsequently underwent multiple logistic regression analyses with the diagnostic discrepancy presence as the dependent variable. We utilised several pre-specified definitions for important variables in the multivariate analyses to affirm our findings, including binary division of the variable AKI (with vs. without) and severity-based grading of AKI (KDIGO grades). Sensitivity analyses based on different age strata and diagnostic subgroups were also conducted. Two-sided *p*-values less than 0.05 were considered statistically significant in all analyses.

## Results

### Clinical features of the participants

We enrolled 188 elderly patients during the study period (21.3% were aged between 60–70 years, 29.3% were between 70–80 years, and 49.5% were older than 80 years), with 84 (44.7%) presenting with AKI upon admission (Table 1). Among those with AKI, 61.9% of cases were related to sepsis, followed by hypotension (14; 16.7%), cardiorenal syndrome (11; 13.1%), dehydration (5; 6%), pancreatitis (1; 1.2%), and hepatorenal syndrome (1; 1.2%).

There was no significant difference between participants with or without initial AKI regarding demographic profiles (age and sex) and comorbidities. The participants with an initial AKI diagnosis had significantly lower systolic and diastolic BP compared to those without (initial AKI vs. without initial AKI: systolic BP, 125 vs. 145 mm Hg, *p* < 0.01; diastolic BP, 72 vs. 80 mm Hg, *p* < 0.01). Half of this cohort was admitted due to cardiopulmonary disorders (49%), followed by gastrointestinal and hepatic disorders (18%), and nephrourological disorders (12%). No significant differences were observed among laboratory data, diagnoses at admission, or receipt of intensive care between those with and without AKI. Among the participants, the overall hospital length of stay was 15.4 ± 15.6 days, and the mortality rate was 12%. Elderly with increasing severity of AKI had a significantly higher mortality (for non-AKI vs. KDIGO stage 1 vs. stage 2 vs. stage 3, 10% vs. 7% vs. 15% vs. 32%, *p* = 0.04) and a trend of longer hospital stay (for non-AKI vs. KDIGO stage 1 vs. stage 2 vs. stage 3, 14.4 ± 11 vs. 13.8 ± 12.5 vs. 19.1 ± 13.8 vs. 21 ± 34.5 days, *p* = 0.21). Upon discharge, the distribution of discharge diagnoses was similar to that of the admission diagnoses (Table 1).

### Diagnostic discrepancy among elderly inpatients

After comparison between the admission and the discharge diagnoses, we found that 28 (14.9%) participants had a diagnostic discrepancy. A description of all the cases with a diagnostic discrepancy is provided in Table 2. One-fourth of the 28 patients were initially admitted for pulmonary/respiratory disorders and FUO, followed by nephrourological disorders (21%), and gastrointestino-hepatic disorders (14%).

**Figure 1:**
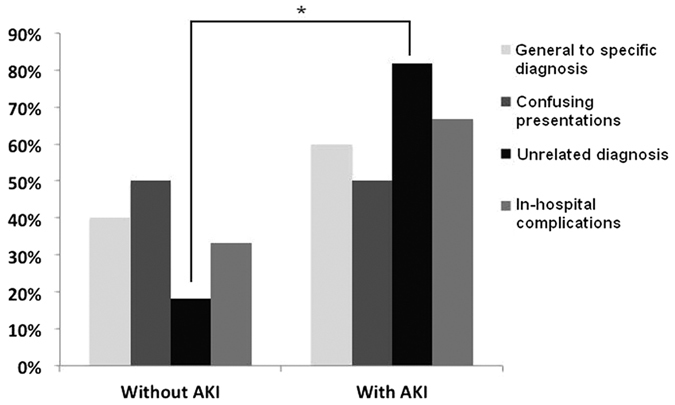
Reasons for the cases with diagnostic discrepancy in the elderly patients *p = 0.01. Abbreviation: AKI, acute kidney injury.

Participants with initial AKI upon admission were significantly more likely to have a diagnostic discrepancy on discharge (with vs. without: 23% vs. 9%, *p* < 0.01). We further classified the diagnostic discrepancies into the following four types based on their reasoning: a switch from a general to a specific diagnosis (n = 10), confusion with presentations of different diseases (n = 4), emergence of in-hospital complications (n = 3), and an unrelated diagnosis not related to in-hospital complications (n = 11). We found that the participants with initial AKI were more likely to have a diagnostic discrepancy from an unrelated diagnosis compared to those without (with vs. without, 82% vs. 18%, *p* = 0.01; Fig. 1).

### Comparisons between participants with and without diagnostic discrepancy

No significant difference in demographic profiles was observed between participants with and without a diagnostic discrepancy (Table 3). Those with a diagnostic discrepancy were significantly more likely to have heart failure (with vs. without: 32% vs. 16%, *p* = 0.04), but not other comorbidities. Nephrourological disorders and FUO were the most common admission diagnoses among the participants with a diagnostic discrepancy compared to those without (*p* = 0.03). Participants with a diagnostic discrepancy were more likely to have an initial AKI diagnosis on admission (*p* < 0.01), more likely to have received intensive care during hospitalization (*p* = 0.01), and had higher mortality (*p* < 0.01). Finally, oncologic disorders, fever of other infectious foci, and non–infection related fever were more common discharge diagnoses among the participants with a diagnostic discrepancy compared to those without (*p* < 0.01).

### Regression analysis for factors influencing diagnostic discrepancies in the participants

We conducted multiple regression analysis to assess the relationship between an initial AKI diagnosis upon admission and diagnostic discrepancy among the participants of this cohort. After accounting for clinical parameters (demographic profiles, comorbidities, admission diagnoses, laboratory data) and treatment course variables, initial AKI diagnosis upon admission was associated with a significantly higher risk of a diagnostic discrepancy on discharge (OR 3.3, *p* < 0.01) (Table 4). An AKI of higher severity was also associated with a stepwise higher risk of a diagnostic discrepancy (for KDIGO grades 1, 2, and 3, OR 2.92, 3.91, and 4.32, *p* = 0.04, 0.03, and 0.02, respectively). These associations remained significant even after adjusting for other clinical parameters (Table 4). We also used different types of sensitivity analysis to affirm the validity of our findings. First, we focused on patients of different age strata for sub-analysis, with the results being essentially similar. Second, we excluded participants with stage 5 CKD (eGFR < 15 ml/min/1.73 m^2^) and repeated the analysis. AKI was still associated with a significantly higher risk of diagnostic discrepancy among the remaining participants (OR 5.38, *p* = 0.002). The participants with increasing AKI severity also exhibited a stepwise higher risk of having a diagnostic discrepancy (OR 4.61, 4.38, and 11.7 for stage 1, 2, and 3 AKI, *p* = 0.01, 0.04, and 0.002, respectively). Finally, we excluded those with FUO for reassurance. AKI was significantly associated with a higher risk of diagnostic discrepancy (OR 7.96, *p* = 0.001). Participants with increasing AKI severity exhibited a stepwise higher risk of diagnostic discrepancy (OR 6.74, 9.03, and 10.3 for stage 1, 2, and 3 AKI, *p* = 0.006, 0.023, and 0.004, respectively). It appears that removing those with advanced CKD or those with FUO further strengthens the identified associations, lending more credibility to our findings.

## Discussion

We found that 14.9% of the participants had a diagnostic discrepancy upon discharge. Those with a diagnostic discrepancy were more likely to have been admitted for a nephrourological disorder or FUO, and no significant difference in comorbidity patterns was found regardless of discrepancy status. The presence of an initial AKI diagnosis on admission was significantly more common among the participants with a diagnostic discrepancy, with a correlation between severity of initial AKI and incrementally higher risk of a diagnostic discrepancy at discharge. Hence, it is likely that an initial AKI diagnosis among geriatric inpatients might interfere with the physicians’ ability to make appropriate diagnoses on admission, which could lead to diagnostic or treatment delay and potentially contribute to adverse patient prognosis.

The incidence of AKI has recently increased with increasing awareness among healthcare workers and patients, as well as the availability of novel biomarkers. AKI predominantly affects the elderly, who have both structural and functional renal alterations and less physiologic reserve to cope with nephrotoxic injuries^17^. Epidemiologic studies have suggested that the risk of AKI has increased by 5- to 10-fold in the elderly compared to that in the general population. The incidence of geriatric AKI varies widely between 5% and 50%, depending on the patients’ age, the clinical setting (medical, surgery, or mixed), and the requirement for intensive care^12^,^18^,^19^. In our cohort, the participants were predominantly of advanced age (mean, 77.9 years), and 44.7% of them had AKI on admission. This number is within the high-normal range of that reported in the literature, and underlines the need for assessing the impact of AKI in the geriatric population.

The issue of diagnostic discrepancy in hospitalised patients is rarely addressed in the literature, with no studies focusing on the elderly. The importance of evaluating diagnostic discrepancy might result from the concern over a close link between diagnostic discrepancies and medical errors^6^. Although the reasons for diagnostic discrepancy include true diagnostic errors, disagreement in interpretation, or inconsistencies in diagnostic criteria, the incidence of diagnostic discrepancies frequently parallels that of medical errors^6^,^20^. Consequently, improving diagnostic accuracy and mitigating factors that contribute to diagnostic discrepancies attenuate error rates and lower the risk of adverse events during hospitalization.

Notably, researchers started describing the prevalence of diagnostic discrepancies and their determinants 4 decades ago; they found that 26.8% of participants had diagnostic changes upon discharge^21^. A more recent study comparing the diagnoses made by emergency physicians to those made upon discharge discovered that 7% to 10% of admissions contained clinically important diagnostic discrepancies^22^. Similarly, Heuer *et al*. reported that nearly 10% of discharge diagnoses differed from the admission diagnoses in a multi-centre study^23^. Based on this, the rate of diagnostic discrepancy has decreased over time, presumably due to the advancement in medical diagnostics and the clinical experience of physicians^24^. In our study, we also observed that 14.9% of the participants had a diagnostic discrepancy after discharge (Table 3), which is slightly higher than that recently reported by other groups. This could result from our focus on geriatric patients only, as the incidence of diagnostic discrepancy is reportedly higher among the elderly and nursing home residents^23^.

The patient characteristics affecting the incidence of diagnostic discrepancy remain unclear. Advanced age, different admission diagnoses (medical diseases as opposed to surgical diseases), neurological disorders, and the presence of neurologic impairment have all been reported to increase the possibility of disagreement between admission and discharge diagnoses^21^,^23^. In addition, autopsy studies comparing postmortem diagnoses to antemortem diagnoses have suggested that patients with admission diagnoses of nephrourological or infectious diseases tend to have a diagnostic discrepancy^9^. Since nearly half of our participants with a diagnostic discrepancy were admitted for these disorders, our findings are similar to those of these autopsy studies (Table 3). We show that elderly participants with a diagnostic discrepancy are more likely to have AKI upon admission, independent of other clinical features, which has not been previously reported. In light of our finding, we propose that more attention is needed in the care of elderly patients with AKI upon admission to avoid potential medical errors during their hospitalization.

The reasons for diagnostic discrepancies include, but are not limited to, a switch from a general diagnosis to a more specific one in relevant fields, grossly incorrect initial impressions due to various reasons, an over- or under-estimation of disease severity, and others^22^,^23^,^25^. We discovered that most elderly patients with a diagnostic discrepancy were found to have unrelated diagnoses (39%), while a switch from general to specific diagnosis accounted for 36% of the cases (Fig. 1). Furthermore, the higher frequency of a diagnostic discrepancy among the elderly with AKI is likely due to a higher proportion of unrelated diagnoses made upon discharge. This is an interesting finding that might explain the association between AKI and diagnostic discrepancies. AKI adversely influences the outcomes of hospitalised patients, and early diagnosis and management of AKI has been strongly suggested by various researchers^26^. Considering the increasing awareness of AKI-related symptomatology, a possibility exists that the emphasis on AKI might overshadow the importance of other symptoms reported by patients, leading to an incomplete initial diagnosis list. Moreover, disease-related complaints of older patients are often more subtle and atypical compared to those of younger patients, further diverting the physician’s attention away from the main illnesses to AKI^27^. This increases the possibility of making an incorrect diagnosis during the initial visit^28^. It is also possible that the symptoms caused by AKI can mislead the physicians and prompt them to make an alternative diagnosis. In addition, the components of the examination for AKI, including serial blood and urine tests, radiologic examinations, or even renal biopsy, might supplant the necessary diagnostic tests for other accompanying illnesses. Moreover, adjunct diagnoses might be overlooked. A more holistic and comprehensive approach for managing geriatric patients is frequently needed, but when a major diagnosis, such as AKI, appears in the list of impressions, the effort to investigate other accompanying diagnoses might be less of a priority. This is an under-recognised influence on medical practice posed by AKI, especially in the elderly. Nonetheless, more investigations are needed to clarify other associations between initial AKI and diagnostic discrepancy in this population.

There are strengths and limitations to this study. Regarding strengths, we have identified an important risk factor; diagnostic discrepancies represent an important but under-recognised tool for assessing the care of the elderly. The dose-dependent relationship between AKI and diagnostic discrepancy further lends to the credibility of our findings. However, the single-centre nature and geriatric-only patient cohort in medical wards limit the applicability of these results. In addition, the size of our cohort is modest; studies enrolling more elderly patients might validate this phenomenon further. Additionally, the characteristics of patients admitted to the general medical wards of this institute might be different from those admitted to others, and extrapolation of our findings might not be feasible. More studies focusing on this issue are required to extend our findings.

## Conclusion

The elderly population is gaining importance in healthcare, given the constant rise in the proportion of the older-aged. Diagnostic discrepancies can assist in the evaluation of diagnostic errors during clinical practice. We found that the incidence of a diagnostic discrepancy was 2- to 3-fold higher among elderly patients with an initial AKI diagnosis on admission, a finding that has not been previously reported. Therefore, reducing the risk of geriatric AKI might lower the incidence of diagnostic discrepancies among this patient population.

## Additional Information

**How to cite this article**: Chao, C.-T. *et al*. Acute kidney injury as a risk factor for diagnostic discrepancy among geriatric patients: a pilot study. *Sci. Rep.* **6**, 38549; doi: 10.1038/srep38549 (2016).

**Publisher's note:** Springer Nature remains neutral with regard to jurisdictional claims in published maps and institutional affiliations.

## Figures and Tables

**Table 1:** Clinical chactersitics of elderly participants recruited in the current study, based on the presence of AKI or not.

Clinical features	Total (n = 188)	With AKI (n = 84)	Without AKI (n = 104)	*p* value
*Demographic profiles*				
Age (years)	77.9 ± 9.7	76.2 ± 9.5	79.3 ± 9.7	0.06
Gender (male %)	95 (51)	47 (56)	48 (46)	0.18
*Comorbidities* (%)				
Hypertension	107 (57)	46 (55)	61 (58)	0.65
Diabetes mellitus	75 (40)	39 (46)	36 (35)	0.12
Cirrhosis	12 (6)	8 (10)	4 (4)	0.11
Coronary artery disease	15 (8)	6 (7)	9 (9)	0.72
Congestive heart failure	34 (18)	19 (23)	15 (14)	0.14
Peripheral vascular disease	12 (6)	3 (4)	9 (9)	0.11
Chronic obstructive pulmonary disease	19 (10)	9 (11)	10 (10)	0.79
Chronic kidney disease	49 (26)	25 (30)	24 (23)	0.28
Rheumatologic disorders	5 (3)	3 (4)	2 (2)	0.48
Cancer	51 (27)	26 (31)	25 (24)	0.27
Peptic ulcer disease	21 (11)	11 (13)	10 (10)	0.44
Old stroke	36 (19)	11 (13)	25 (24)	0.06
Dementia or Parkinsonism	23 (12)	9 (11)	15 (14)	0.47
Hemiplegia	5 (3)	1 (1)	4 (4)	0.27
*Admission Diagnosis* (%)				*0.15*
Cardiopulmonary	92 (49)	36 (43)	56 (54)	
Nephro-urological	22 (12)	15 (18)	7 (7)	
Gastrointestinal and hepatic	34 (18)	18 (21)	16 (15)	
Haemato-oncologic	13 (7)	6 (7)	7 (7)	
Fever of unknown origin	20 (11)	7 (8)	13 (12)	
Cerebrovascular accident	7 (4)	2 (2)	5 (5)	
*Vital signs at presentation*				
Systolic blood pressure (mmHg)	135 ± 36	125 ± 35	145 ± 38	<0.01
Diastolic blood pressure (mmHg)	76 ± 20	72 ± 19	80 ± 20	<0.01
Heart rate (/min)	97 ± 21	98 ± 24	96 ± 19	0.57
*Laboratory parameters*				
White blood cells (K/μL)	12.1 ± 6.1	13.1 ± 7.5	11.3 ± 5.3	0.07
Haemoglobin (g/dL)	11.5 ± 7.5	11 ± 2.8	12.4 ± 10.1	0.12
Platelet (K/μL)	229 ± 108	219 ± 118	236 ± 108	0.31
Baseline creatinine (mg/dL)	1.8 ± 2.4	1.7 ± 2.3	1.9 ± 2.4	0.63
*Treatment courses*				
Care in intensive care units (%)	3 (2)	(1)	(2)	0.7
*Discharge diagnosis* (%)				*0.22*
Cardiopulmonary	90 (48)	33 (39)	57 (55)	
Nephro-urological	21 (11)	14 (17)	7 (7)	
Gastrointestinal and hepatic	32 (17)	17 (20)	15 (14)	
Haemato-oncologic	19 (10)	9 (11)	10 (10)	
Fever of other infection foci or non-infection related	17 (9)	8 (10)	9 (9)	
Cerebrovascular accident	9 (5)	3 (4)	6 (6)	

Data are expressed as mean ± standard deviation for continuous variables, and number (percentage) for categorical variables.

**Table 2:** The list of cases with diagnostic discrepancy among the entire cohort.

Admission diagnosis	Discharge diagnosis	Event (percentage)
*Pulmonary and respiratory tract disorders*		
Pneumonia	Lung cancer, heart failure, urinary tract infection	6 (21)
Acute tracheobronchitis	Urinary tract infection	1 (4)
*Fever of unknown origin*	*Pneumonia, ischaemic stroke, ileus, urinary tract infection, infective endocarditis*	*7 (25*)
*Nephro-urological disorders*		
Urinary tract infection	Urothelial carcinoma with multiple metastasis, pneumonia, ilues	5 (18)
Acute kidney injury	SIADH	1 (4)
*Gastrointestinal and Hepatic disorders*		
Ileus	Colon adenocarcinoma	1 (4)
Biliary tract infection	Colostomy with peri-stomal abscess	1 (4)
Intra-abdominal infection	Acute lymphoid leukaemia	1 (4)
Duodenal stenosis	Urothelial carcinoma	1 (4)
*Cardiovascular disorders*		
Congestive heart failure	Pneumonia, chronic obstructive pulmonary disease	2 (7)
Peripheral artery occlusive disease	Hypovolaemic and septic shock	1 (4)
*Oncologic disorders*		
Gallbladder cancer with multiple metastasis	Pneumonia	1 (4)

Abbreviation: SIADH, syndrome of inappropriate anti-diuretic hormone.

**Table 3:** Comparison of elderly patients with and without diagnostic discrepancies.

Clinical features	With (n = 28)	Without (n = 160)	*p* value
*Demographic profiles*			
Age (years)	76.4 ± 9.9	78.2 ± 9.7	0.36
Gender (male %)	10 (36)	85 (53)	0.09
*Comorbidities* (%)			
Hypertension	11 (39)	65 (41)	0.19
Diabetes mellitus	19 (68)	88 (55)	0.91
Cirrhosis	1 (4)	11 (7)	0.52
Coronary artery disease	2 (7)	13 (8)	0.87
Congestive heart failure	9 (32)	25 (16)	0.04
Peripheral vascular disease	0 (0)	12 (8)	0.12
Chronic obstructive pulmonary disease	1 (4)	18 (11)	0.22
Chronic kidney disease	11 (39)	38 (24)	0.08
Rheumatologic disorders	3 (11)	2 (1)	0.13
Cancer	6 (21)	45 (28)	0.48
Peptic ulcer disease	5 (18)	16 (10)	0.22
Old stroke	4 (14)	32 (20)	0.45
Dementia or Parkinsonism	4 (14)	19 (12)	0.79
Hemiplegia	1 (4)	4 (3)	0.74
*Admission Diagnosis* (%)			*0.03*
Cardiopulmonary	10 (36)	82 (51)	
Nephro-urological	6 (21)	16 (10)	
Gastrointestinal or hepatic	4 (14)	30 (19)	
Oncologic	1 (4)	12 (8)	
Fever of unknown origin	7 (25)	13 (8)	
Cerebrovascular accident	0 (0)	7 (4)	
*Vital signs at presentation*			
Systolic blood pressure (mmHg)	132 ± 27	136 ± 38	0.58
Diastolic blood pressure (mmHg)	74 ± 15	77 ± 20	0.48
Heart rate (/min)	100 ± 21	97 ± 20	0.45
*Laboratory parameters*			
White blood cells (K/μL)	13.5 ± 7.4	11.8 ± 5.9	0.18
Haemoglobin (mg/dL)	10.2 ± 2.2	11.8 ± 8.1	0.3
Platelet (K/μL)	232 ± 116	228 ± 107	0.86
Baseline creatinine (mg/dL)	2.2 ± 2.7	1.7 ± 2.3	0.34
*Initial AKI on presentation*	*19 (68*)	*65 (41*)	<*0.01*
*Treatment courses*			
Care in intensive care units (%)	2 (7)	1 (1)	0.01
Hospital outcomes (death %)	8 (29)	15 (9)	0.01
*Discharge diagnosis* (%)			*0.01*
Cardiopulmonary	10 (36)	81 (50)	
Nephro-urological	4 (14)	17 (11)	
Gastrointestinal or hepatic	1 (4)	31 (19)	
Oncologic	5 (18)	14 (9)	
Fever of other infection foci or non-infection related	6 (21)	11 (7)	
Cerebrovascular accident	2 (7)	7 (4)	

Data are expressed as mean ± standard deviation for continuous variables, and number (percentage) for categorical variables.

Abbreviation: AKI, acute kidney injury.

**Table 4:** Multiple logistic regression analyses with diagnostic discrepancy as the dependent variable.

Results	Odds ratio	95% Confidence Interval	*p* value
*Model 1*			
Congestive heart failure	2.25	0.89–5.7	0.09
Initial AKI or not	3.3	1.37–8	<0.01
*Model 2*			
AKI KDIGO grade 1	2.92	1.06–8.09	0.04
AKI KDIGO grade 2	3.91	1.13–13.6	0.03
AKI KDIGO grade 3	4.32	1.23–15.2	0.02
*Model 3*			
Congestive heart failure	2.48	0.93–6.64	0.07
Initial AKI or not	3.52	1.38–9	<0.01
*Model 4*			
AKI KDIGO grade 1	2.92	1.06–8.09	0.04
AKI KDIGO grade 2	3.91	1.13–13.6	0.03
AKI KDIGO grade 3	4.32	1.23–15.2	0.02

Model 1 included variables from demographic profiles (age and gender), comorbidities, admission diagnoses, the presence of AKI, and ICU care.

Model 2 included variables from demographic profiles (age and gender), comorbidities, admission diagnoses, the severity of AKI if presence (by KDIGO grading), and ICU care.

Model 3 included model 1 variables and vital signs at presentation.

Model 4 included model 2 variables and vital signs at presentation.

Abbreviations: AKI, acute kidney injury; ICU, intensive care unit; KDIGO, Kidney Disease Improving Global Outcomes.
